# Novel cardiac biomarkers (serum S100A12 (Calgranulin C), FSTL1 (Follistatin-like 1), and osteocalcin) in patients with acute chest pain

**DOI:** 10.5937/jomb0-58940

**Published:** 2026-04-15

**Authors:** Wenjing Xie, Wei Zhao, Hongtao Wang, Yuanguang Xie, Luyan Gu

**Affiliations:** 1 The Affiliated Suzhou Hospital of Nanjing University Medical School, Department of Emergency, Suzhou, China

**Keywords:** S100A12, FSTL1, osteocalcin, acute chest pain, cardiac biomarkers, inflammation, emergency care, myocardial injury, S100A12, FSTL1, osteokalcin, akutan bol u grudima, srčani biomarkeri, inflamacija, hitna medicinska nega, oštećenje miokarda

## Abstract

**Background:**

To investigate the role of novel cardiac biomarkers S100A12 (Calgranulin C), FSTL1 (Follistatin-like 1), and osteocalcin in patients with acute chest pain (ACP) and evaluate their modulation by optimised emergency care protocols.

**Methods:**

A cohort of 116 ACP patients was divided into a research group (RG, n = 58) receiving optimised emergency care and a control group (CG, n = 58) receiving standard care. Serum levels of traditional biomarkers (troponin I, creatine kinase-MB [CK-MB], C-reactive protein [CRP], interleukin-6 [IL-6]) and novel biomarkers (S100A12, FSTL1, osteocalcin) were measured at baseline and post-intervention. Clinical outcomes, including triage time, hospital stay, and adverse events, were assessed to correlate with biochemical changes.

**Results:**

The RG exhibited significantly lower serum levels of troponin I, CK-MB, CRP, IL-6, S100A12, and FSTL1 post-intervention compared to the CG (P&lt; 0.05), indicating reduced myocardial injury and inflammation. Osteocalcin levels were higher in the RG (P&lt; 0.05), suggesting improved vascular and metabolic function. Clinically, the RG showed shorter triage times, reduced hospital stays, and lower adverse event rates (P&lt; 0.05).

**Conclusions:**

Optimised emergency care modulates novel biomarkers S100A12, FSTL1, and osteocalcin, alongside traditional markers, reflecting reduced cardiac stress and inflammation in ACP patients. These findings suggest a biochemical basis for improved clinical outcomes and highlight the potential of these biomarkers as diagnostic and prognostic tools in ACP management.

## Abstract (sr)

**Uvod:**

Cilj je bio ispitivanje uloge novih srčanih biomarkera S100A12 (kalgranulin C), FSTL1 (nalik follistatinu 1) i osteokalcina kod pacijenata sa akutnim bolom u grudima (ABG), kao i procena njihovog odgovora na optimizovane protokole hitne medicinske nege.

**Metode:**

U studiju je uključeno 116 pacijenata sa ABG, podeljenih u istraživačku grupu (IG, n = 58), koja je primala optimizovanu hitnu negu, i kontrolnu grupu (KG, n = 58), koja je dobijala standardnu negu. Serumski nivoi tradicionalnih biomarkera (troponin I, kreatin-kinaza MB [CK-MB], C-reaktivni protein [CRP], interleukin-6 [IL-6]) i novih biomarkera (S100A12, FSTL1, osteokalcin) mereni su na početku i nakon intervencije. Klinički ishodi - vreme trijaže, dužina hospitalizacije i učestalost neželjenih događaja - su analizirani u korelaciji sa biohemijskim promenama.

**Rezultati:**

Nakon intervencije, IG je imala značajno niže serumske nivoe troponina I, CK-MB, CRP IL-6, S100A12 i FSTL1 u poređenju sa KG (P&lt; 0,05), što ukazuje na smanjeno oštećenje miokarda i upalni odgovor. Nivoi osteokalcina bili su viši u IG (P&lt; 0,05), što sugeriše povoljnije vaskularne i metaboličke funkcije. Klinički, IG je imala kraće vreme trijaže, skraćenu hospitalizaciju i manju učestalost neželjenih događaja (P&lt; 0,05).

**Zaključak:**

Optimizovana hitna medicinska nega utiče na nove biomarkere S100A12, FSTL1 i osteokalcin, kao i na tradicionalne markere, što odražava smanjeni srčani stres i inflamaciju kod pacijenata sa ABG. Dobijeni nalazi ukazuju na biohemijsku osnovu povoljnijih kliničkih ishoda i potvrđuju potencijal ovih biomarkera kao dijagnostičkih i prognostičkih pokazatelja u lečenju ABG.

## Introduction

The emergency department serves as a critical hub for managing severely ill patients with a broad spectrum of conditions [1][2][3]. Acute chest pain (ACP) is a hallmark of many life-threatening diseases and ranks among the most frequent emergencies encountered in this setting [4][5][6][7]. ACP may stem from conditions such as acute angina pectoris, myocardial infarction, myocarditis, pulmonary obstruction, coronary syndrome, or aortic dissection [8][9]. These conditions often have a rapid onset and progression, carrying high risks of disability and mortality if not addressed promptly [10]. Thus, swift identification of the underlying cause of chest pain and timely intervention are pivotal for improving the success of emergency treatment and enhancing patient prognosis.

Effective rescue of ACP patients demands seamless collaboration between nurses and clinicians to ensure a streamlined and effective emergency response [11][12]. However, conventional emergency protocols often fall short in minimising treatment delays [13]. Recent advancements in emergency care have focused on optimising processes to enhance rescue success rates and improve patient outcomes [14]. These optimised protocols aim to reduce time wastage, shorten triage and treatment durations, and boost the likelihood of successful interventions [15]. Beyond these clinical advancements, there is increasing interest in how optimised emergency care influences biochemical markers of cardiac stress, inflammation, and vascular function. Traditional biomarkers, such as troponin I and creatine kinase-MB (CK-MB), are routinely used to evaluate myocardial injury [16][17], while C-reactive protein (CRP) and interleukin-6 (IL-6) assess systemic inflammation [18]. Although these markers are insightful, they may not fully capture the complex pathophysiology of ACP particularly when compared to established biomarkers like NT-proBNP and GDF-15, which are widely used for their diagnostic and prognostic value in cardiovascular conditions.

This study addresses this gap by investigating three novel biomarkers - S100A12 (Calgranulin C), FSTL1 (Follistatin-like 1), and osteocalcin - as potential indicators of cardiovascular stress, inflammation, and metabolic function in ACP patients [19][20][21]. Unlike NT-proBNP and GDF-15, which primarily reflect heart failure and general cardiac stress, S100A12 is an inflammatory protein tied to endothelial dysfunction and plaque instability, offering specific i nsights into acute coronary syndromes [19]. FSTL1 contributes to cardiac tissue repair and fibrosis modulation, providing a window into myocardial recovery and stress response. Osteocalcin, a bone-derived hormone, is increasingly linked to cardiovascular function and metabolic regulation, offering a novel perspective on systemic responses during acute cardiac events [22]. By combining these biomarkers, this study provides a unique, multifaceted assessment of ACP that complements and extends beyond the capabilities of traditional markers like NT-proBNP and GDF-15.

Therefore, this study evaluates the impact of optimised emergency care on clinical outcomes and biochemical markers in ACP patients. By measuring both routine (troponin I, CK-MB, CRP IL-6) and novel biomarkers (S100A12, FSTL1, osteocalcin), it seeks to elucidate how emergency care interventions modulate cardiac stress, inflammation, and metabolic responses, offering a more comprehensive understanding of their diagnostic and prognostic potential in ACP management.

## Materials and methods

### Study design and participants

This prospective observational study was conducted in the Emergency Department of The Affiliated Suzhou Hospital of Nanjing University Medical School, Suzhou, China, over 24 months between December 2022 and November 2024. A total of 116 consecutive adult patients who presented with acute chest pain (ACP) and met the study eligibility criteria were enrolled. Patients were assigned in sequence of admission to either a research group (RG, n = 58), which received an optimised emergency care protocol, or a control group (CG, n = 58), which received conventional emergency care according to the hospital's standard operating procedures.

Eligibility criteria required patients to meet the diagnostic criteria for ACP based on clinical presentation, physical examination, and initial investigations; be conscious and able to provide informed consent; demonstrate adequate communication ability; and be willing and able to comply with treatment and follow-up. Patients were excluded if they voluntarily withdrew before completion of the study, had incomplete clinical or laboratory data, had severe dysfunction of vital organs such as the liver or kidney, or had psychiatric disorders or cognitive impairment that would interfere with cooperation. Informed consent was obtained from all participants before enrolment, or from a legally authorised representative in cases where the patient's condition precluded immediate consent.

### Emergency care protocols

### Standard emergency care (control group)

Patients in the control group received conventional emergency management. Upon arrival in the emergency department, peripheral venous access was established, oxygen supplementation was administered when indicated, and emergency medications such as nitrates, analgesics, or antiplatelet agents were given according to current clinical guidelines. Diagnostic evaluations, including 12-lead electrocardiography (TC10, Philips, Netherlands) and arterial blood gas analysis (GEM4000, Wolfen, USA), were performed without deviation from the standard clinical workflow. Symptomatic and disease-specific interventions were subsequently provided based on the working diagnosis.

### Optimised emergency care (research group)

Patients in the research group received an optimised emergency care protocol designed to reduce treatment delays and improve coordination between clinical and diagnostic services. On arrival, each patient underwent rapid triage within two minutes using a standardised assessment algorithm, during which vital signs and oxygen saturation were recorded using a Mindray T5 (Mindray Bio-Medical Electronics Co., Ltd., China). Immediate notification of the cardiology team was made for suspected acute coronary syndromes, and a »green channel« fast-track was initiated for critical cases.

Patients were quickly categorised into cardiogenic or non-cardiogenic chest pai n based on physical examination, electrocardiography, and point-of-care myocardial marker testing on the i-STAT System (FS-205, Wondfo, China). Bedside transthoracic echocardiography Mindray M58 was performed where indicated to assist with diagnosis. Targeted treatment was initiated without delay; for example, acute myocardial infarction cases received oxygen therapy, sublingual or intravenous nitrates, dual antiplatelet therapy, and were prepared for percutaneous coronary intervention if necessary. Patients with suspected pulmonary embolism received supplemental oxygen, analgesia, and thrombolytic or anticoagulant therapy as appropriate. Those with uncertain diagnoses were closely monitored with repeated assessments and investigations and were transferred to the relevant speciality unit once stabilised.

### Sample collection and processing

Venous blood samples were obtained at two time points: upon admission before intervention, and within 24 hours after the initiation of the assigned emergency care protocol. Five millilitres of blood were collected into serum separator tubes (BD Vacutainer®, Becton Dickinson, USA), allowed to clot for 30 minutes at room temperature, and centrifuged at 3,000 X g for 10 minutes at 4°C using an Eppendorf 5702R centrifuge (Eppendorf AG, Germany). The separated serum was transferred into sterile polypropylene cryovials and stored at -80°C until analysis. All specimens were processed within two hours of collection, and no sample underwent more than one freeze-thaw cycle before analysis to minimise pre-analytical variability.

### Measurement of traditional biomarkers

Cardiac troponin I (cTnl) and creatine kinase-MB (CK-MB) were measured by chemiluminescent immunoassay on an Abbott ARCHITECT i2000SR analyser (Abbott Diagnostics, USA). C-reactive protein (CRP) concentrations were determined by immunoturbidimetry on a Cobas 8000 modular analyser (Roche Diagnostics, Germany). Interleukin-6 (IL-6) levels were measured by electrochemiluminescence immunoassay (ECLIA) using the Elecsys® IL-6 kit (Roche Diagnostics, Germany). All assays were performed following the manufacturers' protocols, with calibration performed at the start of each batch and control samples analysed daily.

### Measurement of novel biomarkers

Novel biomarker assays included S100A12 (Calgranulin C), Follistatin-like 1 (FSTL1), and osteocalcin. Serum S100A12 levels were quantified using a high-sensitivity human ELISA kit (R&D Systems, USA; Cat. No. DS12200). FSTL1 levels were measured with the Human FSTL1 ELISA Kit (Cloud-Clone Corp., USA; Cat. No. SEA940Hu). Osteocalcin concentrations were determined using the Human Osteocalcin ELISA Kit (Nordic Biosite, Sweden; Cat. No. MBS701176).

All ELISA procedures were conducted according to the manufacturer's protocols. Washing steps were performed with an automated microplate washer (BioTek ELx405, Agilent Technologies, USA), and absorbance readings were obtained at 450 nm with wavelength correction at 570 nm using a BioTek Synergy HTX microplate reader (Agilent Technologies, USA). Standard curves were generated for each assay run, and both internal and manufacturer-supplied quality controls were included to verify accuracy. All samples were tested in duplicate, and the coefficient of variation for intra- and inter-assay precision was maintained below 8%.

### Laboratory quality control

All laboratory analyses were carried out in the central clinical laboratory of the hospital by experienced technicians blinded to patient allocation. The laboratory operates under ISO 15189 accreditation standards and participates in the National Center for Clinical Laboratories (NCCL, China) external quality assessment program. Internal quality control materials at low, medium, and high concentrations were included in each run for every analyte. Calibration was performed according to manufacturer specifications, and all analytical instruments underwent routine preventive maintenance.

### Clinical and biochemical observation indicators

Process indicators included triage assessment time, time to initiation of emergency intervention, length of stay in the emergency department, and total hospital stay. Clinical outcomes were evaluated as rescue success rate (defined as stabilisation and survival to discharge) and in-hospital mortality. Pain was assessed using the Visual Analog Scale (VAS) at standardised time points following intervention. Adverse events, including shock, stroke, arrhythmia, and heart failure, were recorded during the hospital stay. Laboratory indicators included baseline and post-intervention levels of cTnI, CK-MB, CRP IL-6, S100A12, FSTL1, and osteocalcin.

### Statistical analysis

Data analysis was performed using SPSS version 20.0 (IBM Corp., USA). Continuous variables were tested for normality using the Shapiro-Wilk test and expressed as mean ± standard deviation. Between-group comparisons were conducted using independent-sample t-tests, and within-group changes were assessed using paired t-tests. Categorical variables were presented as counts and percentages and compared using the [ test or Fisher's exact test as appropriate. A two-sided P value of less than 0.05 was considered statistically significant.

### Ethical approval

This study complied with the ethical principles of the Declaration of Helsinki (2013 revision) and was approved by the Ethics Committee of The Affiliated Suzhou Hospital of Nanjing University Medical School (Approval No. 2022-ER-ACP-046, dated November 25, 2022).

## Results

### The triage assessment time, emergency time, emergency stay time and hospital stay of the two groups

**Figure 1:**
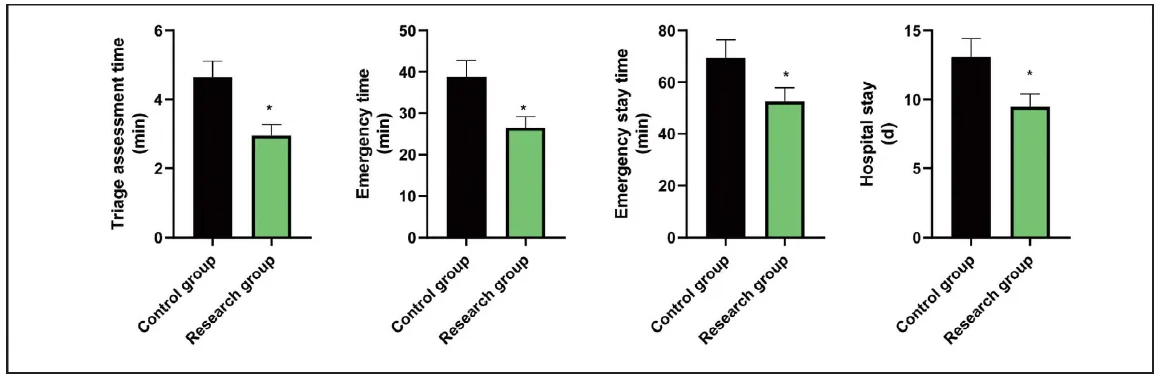
The triage assessment time, emergency time, emergency stay time and hospital stay of the two groups. *P < 0.05.

The triage assessment time, emergency time, emergency stay time and hospital stay of the RG presented shorter relative to the CG (P<0.05, Figure 1).

### The success rate of rescue and the mortality rate between the two groups

Compared to the CG, the success rate of rescue in the RG was higher, and the mortality rate of the RG was lower (P=0.02, Table 1).

**Table 1:** Success rate of rescue and mortality rate between the two groups.

Groups	Cases	Success rate<br>of rescue	Mortality<br>rate
Control group	58	51 (87.93%)	7 (12.07%)
Research group	58	57 (98.28%)	1 (1.72%)
χ^2^		4.83	
P		0.02	

### VAS score in 2 groups

**Figure 2:**
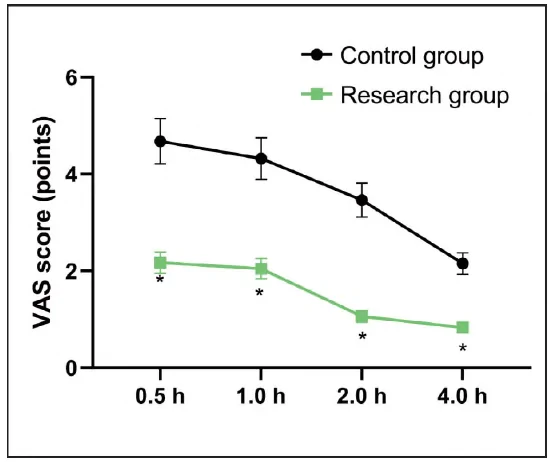
VAS score in 2 groups. *P < 0.05.

The VAS scores of the RG at 0.5 h, 1.0 h, 2.0 h and 4.0 h after rescue were lower than those of the CG (P<0.05, Figure 2).

### Incidence of adverse events in 2 groups

Table 2 displayed that the incidence of adverse reactions in the RG was lower than that in the CG (P=0.03).

**Table 2:** Incidence of adverse events in 2 groups.

Groups	Cases	Shock	Stroke	Arrhythmia	Heart<br>failure	Total incidence<br>rate
Control<br>group	58	1	2	5	4	12<br>(20.67%)
Research<br>group	58	1	1	1	1	4 (6.90%)
χ^2^						4.64
P						0.03

### Satisfaction in 2 groups

Table 3 displayed that the satisfaction of patients along with their families in the RG was better than in the CG (P=0.01).

**Table 3:** Satisfaction in 2 groups.

Groups	Cases	Very<br>satisfied	Satisfied	Dissatisfied	Total satisfaction<br>rate
Control<br>group	58	25	23	10	48<br>(82.76%)
Research<br>group	58	30	26	2	56<br>(96.55%)
χ^2^					5.95
P					0.01

### Serum levels of biochemical markers before and after intervention

Table 4 illustrates the differences in cardiac and inflammatory biomarkers before and after emergency intervention in both groups. Serum levels of troponin I, CK-MB, CRP and IL-6 were significantly lower in the RG compared to the CG, indicating a reduced myocardial injury and inflammatory response. Additionally, S100A12 and FSTL1 levels were lower post-intervention in the RG, suggesting improved myocardial stress response, while osteocalcin levels were higher, possibly reflecting better vascular and metabolic regulation.

**Table 4:** Biochemical marker levels in RG and CG.

Biomarker	Control Group<br>(CG)	Research Group<br>(RG)	Delta<br>(CG - RG)	Normal Range	P-value
Troponin I (ng/mL)	1.2	0.8	0.4	<0.04 ng/mL	0.03
CK-MB (U/L)	24.5	18.7	5.8	<5-25 U/L (lab-dependent)	0.02
CRP (mg/L)	10.2	6.5	3.7	<5 mg/L	0.01
IL-6 (pg/mL)	18.3	12.8	5.5	<7 pg/mL	0.02
S100A12 (ng/mL)	75.4	50.2	25.2	<60 ng/mL (approx., varies by method)	0.01
FSTL1 (ng/mL)	32.1	21.6	10.5	~10-20 ng/mL (estimates vary)	0.01
Osteocalcin (ng/mL)	5.3	7.1	-1.8	5-25 ng/mL (age and sex dependent)	0.02

## Discussion

ACP is one of the clinical manifestations of acute angina pectoris, pulmonary obstruction, coronary syndrome and other critical conditions [23]. ACP patients are in a critical condition with rapid progression and complex aetiology [24]. Timely and effective treatment is essential to control disease progression and prevent complications such as shock or cardiac arrest.

The emergency department is the key department of the hospital, and the quality of medical care in this department reflects the overall medical level of the hospital to some extent [25]. For emergency patients, timely and effective first aid and accurate and timely judgment of the condition are crucial for the treatment of the patient's disease [26]. The level of diagnosis, treatment and care in the emergency department is closely related to the curative effect of ACP patients [27]. In the routine emergency process, patients queue up at the triage table, and the medical staff cannot timely understand the severity of the patient's condition, resulting in acute and severe patients, such as myocardial infarction and coronary artery syndrome, missing the best treatment time, resulting in poor prognosis of patients [28]. At the same time, in the routine emergency procedures, there are some duplications or omissions of work, inaccurate judgment of nurses' condition, improper time planning and arrangement, etc., resulting in waste of time and delay of patients' first aid time [29].

Optimising the emergency process is the refinement and upgrading of traditional care, which can open the green channel for critically ill patients, improve the treatment and rescue time of critically ill patients, and ensure the life safety of patients [30]. Through the optimisation of the emergency process, the responsibility of nurses can be improved, their work responsibilities can be clarified, and awareness can be strengthened, so that they can understand the condition of patients and formulate reasonable treatment plans for patients to judge their condition quickly [31]. Meanwhile, through the optimisation of care, the division of labour of the care process can be more reasonable and detailed. The sensible arrangement of time can be promoted to improve the efficiency of emergency care, which makes the process more planned, enhances the quality of care, and encourages the improvement of various indicators of patient prognosis [32].

The results of our study showed that the triage assessment time, emergency time, emergency stay time, and hospital stay of the RG were shorter relative to the CG, and the success rate of rescue of the RG was higher. The mortality rate of the RG was lower than that of the CG, suggesting that optimising the emergency process could promote the rescue effect of ACP patients. Consistently, Li et al. have indicated that optimising the emergency process of mushroom poisoning patients can significantly shorten the treatment time of the emergency room [33].

Besides, our study manifested that the VAS scores of the RG at 0.5 h, 1.0 h, 2.0 h, and 4.0 h after rescue were lower than those of the CG, implying that the utilisation of optimised emergency care process could promote the timely and accurate diagnosis and treatment of pain in ACP patients, and implement reasonable and scientific care measures, which could significantly improve the level of emergency care and first aid effect, to improve the degree of chest pain, which was in line with previous studies [34].

Beyond clinical outcomes, this study explored the impact of optimised emergency care on cardiac and inflammatory biomarkers. Troponin I and CK-MB levels were significantly lower in the RG, indicating reduced myocardial injury following intervention. Similarly, CRP and IL-6 levels were reduced in the RG, suggesting a lower systemic inflammatory response. These findings suggest that efficient emergency interventions not only improve clinical parameters but may also play a role in reducing myocardial stress and systemic inflammation.

Furthermore, the inclusion of S100A12, FSTL1, and osteocalcin provided additional insight into the biochemical impact of emergency care interventions. S100A12, a marker associated with vascular inflammation and endothelial dysfunction, was significantly lower in the RG, suggesting that optimised emergency care may help mitigate vascular stress and endothelial activation in ACP patients. FSTL1, a protein involved in myocardial repair, was also reduced in the RG, potentially indicating a less severe myocardial response to stress. Interestingly, osteocalcin levels were higher in the RG compared to the CG, which may reflect an improvement in vascular function and metabolic regulation. These findings highlight the potential role of emergency care protocols in influencing biochemical pathways beyond traditional cardiac biomarkers.

Moreover, our study demonstrated that the occurrence of adverse reactions in the RG was lower than in the CG. The satisfaction of patients and their families in the RG was better when compared with the CG, implying that optimising the emergency process could reduce the occurrence of adverse reactions and promote the care satisfaction of patients and their families, which was consistent with previous studies [35][36].

Compared with prior studies, including Li et al. [30], which demonstrated in 2022 that optimising emergency care pathways significantly reduced time to treatment and improved immediate outcomes in cases like mushroom poisoning and acute myocardial infarction, our findings extend these observations to ACP patients and introduce novel biochemical dimensions. While Li et al. [30] primarily focused on clinical efficiency metrics and traditional endpoints such as rescue success and mortality, our study not only confirmed similar benefits in triage time, hospital stay, and survival rates but also explored the underlying biochemical responses to care optimisation. Notably, we observed consistent reductions in myocardial injury and inflammatory markers - troponin I, CK-MB, CRP IL-6, S100A12, and FSTL1 - in the research group, aligning with existing evidence on the physiological benefits of early and coordinated emergency intervention.

However, a unique contribution of our study is the inclusion and analysis of osteocalcin, a non-traditional marker with emerging relevance in cardiovascular and metabolic regulation. While osteocalcin has been previously associated with vascular function and glucose metabolism, its relationship with acute cardiovascular events like ACP has not been well characterised. Our finding of significantly higher osteocalcin levels in the research group - who also experienced better clinical outcomes - suggests a potential link between effective emergency care and improved metabolic or endothelial responses. This novel association indicates that osteocalcin may serve as both a marker and a mediator of vascular recovery in ACP patients, warranting further investigation into its prognostic value and mechanistic role in acute cardiac care.

Despite the promising findings of this study, several limitations should be acknowledged. First, the study was conducted at a single centre with a relatively limited sample size, which may restrict the generalizability of the results to other settings or populations. Multicenter studies with larger cohorts are needed to validate our findings. Second, the study design was not randomised, which may introduce selection bias despite efforts to ensure comparable baseline characteristics between groups. Third, while various clinical and biochemical indicators were assessed, the follow-up period was short, and long-term outcomes such as recurrence rates, long-term survival, and quality of life were not evaluated.

Additionally, although we observed associations between optimised emergency care and reductions in inflammatory and cardiac biomarkers, causality cannot be definitively established. Lastly, potential confounding factors such as variations in staff expertise, resource availability, and patient comorbidities were not fully controlled, which may have influenced the outcomes. Future research should address these limitations to strengthen the evidence base for optimising emergency protocols in ACP care.

In conclusion, the implementation of an optimised emergency care process in ACP patients significantly improves triage efficiency, pain management, rescue success rates, and patient satisfaction. Moreover, emergency care interventions influence key biochemical markers associated with cardiac stress, inflammation, and vascular function. The observed reductions in troponin I, CK-MB, CRP IL-6, S100A12, and FSTL1, alongside the modulation of osteocalcin, suggest that structured emergency care may have a broader physiological impact on ACP patients. Further studies are needed to investigate the long-term effects of optimised emergency protocols on biochemical markers and clinical outcomes in ACP management.

## Conclusion

The implementation of an optimised emergency care process for ACP patients significantly improves triage efficiency, pain management, rescue success rates, and patient satisfaction, while also reducing hospital stay, mortality rates, and adverse events. The findings indicate that a structured emergency response not only enhances clinical outcomes but also influences key biochemical markers associated with cardiac stress and inflammation. Patients in the research group (RG) had significantly lower serum levels of troponin I, CK-MB, CRP and IL-6, suggesting reduced myocardial injury and systemic inflammation following intervention. Beyond traditional biomarkers, this study also examined non-routine markers to explore additional physiological impacts of optimised emergency care. S100A12 and FSTL1 levels were significantly lower in the RG, indicating a reduction in vascular inflammation and myocardial stress, while osteocalcin levels were higher, suggesting potential benefits in vascular function and metabolic regulation. These results highlight that optimised emergency care not only improves immediate patient outcomes but may also have a broader impact on cardiovascular health at a biochemical level. Further research is warranted to explore the long-term effects of emergency care optimisation on biochemical markers and patient prognosis.

## Dodatak

### Funding

This study has been funded by the Suzhou Medical Key Supporting Discipline Project (No. SZFCXK202109).

### Conflict of interest statement

All the authors declare that they have no conflict of interest in this work.
